# Mesenteric lymph nodes are required for B- but dispensable for local T cell effector responses following *Citrobacter rodentium* infection in mice

**DOI:** 10.3389/fimmu.2026.1812877

**Published:** 2026-05-11

**Authors:** Ole Riemer, Jonathan Gehring, Pia Pascale Peppermüller, Mandy Holzberger, Anja Siebert, Marijana Basic, Matthias Lochner, André Bleich, Martin Meier, Manuela Buettner

**Affiliations:** 1 Institute for Laboratory Animal Science, Hannover Medical School, Hannover, Germany; 2 Institute of Medical Microbiology and Hospital Epidemiology, Hannover Medical School, Hannover, Germany

**Keywords:** B cell response, *Citrobacter rodentium* (*C. rodentium*), gut immunity, immune homeostasis, mesenteric lymph nodes (mLN), T cell response

## Abstract

**Introduction:**

The intestinal immune system is organized into regionally specialized lymphatic drainage networks that coordinate adaptive responses according to the anatomical site of microbial encounter. During *Citrobacter rodentium* (*C. rodentium*) infection in mice, initial colonization occurs in the cecum and proximal colon, where antigen‑presenting cells drain to the corresponding mesenteric lymph nodes (mLN), before the pathogen progresses to dominant attachment in the distal colon at peak infection. These segment‑specific patterns of colonization are matched by segment‑specific lymphatic drainage, such that distinct lymph node compartments support immune priming in different intestinal regions. Understanding how these proximal draining mLN contribute to the development of colonic immunity, despite the later dominance of distal‑colonic infection, remains an important open question.

**Methods:**

To examine the contribution of the colon‑draining mLN, we surgically removed either the entire mLN chain or only the nodes draining the cecum and colon, followed by *C. rodentium infection*. Immune responses were analyzed at day 10 and day 18, corresponding to phases in which distal‑colonic colonization dominates.

**Results:**

We found that Th17 effector responses in the colon were maintained independently of the removed mLN, consistent with the fact that local colonic activation is the primary driver of the observed T cell phenotype at these time points. In contrast, B cell differentiation was markedly impaired in mLN‑resected animals: both plasma cell frequencies in the colon and pathogen‑specific serum IgG1 and IgG2a responses were significantly reduced.

**Discussion:**

These findings indicate that, although distal‑colonic T cell activation proceeds locally, effective B cell activation and class switching depend on the presence of the appropriate draining lymph nodes. Overall, this study highlights the segment‑specific organization of intestinal immunity and demonstrates that B cell responses during *C. rodentium* infection are critically dependent on the lymph nodes draining the cecum and colon, whereas local T cell activation in the distal colon can occur independently of these structures.

## Introduction

Inflammatory intestinal diseases caused by pathogenic bacteria are a widespread and clinically relevant health issue. The mouse model of *Citrobacter rodentium* (*C. rodentium*) infection is frequently used to gain a better understanding of the underlying mechanisms. This model is well established and allows detailed investigations into the pathogenesis and the host’s immune response to enteral infections (1, 2).

*C. rodentium* infection closely resembles infections with human pathogenic bacteria such as enterohemorrhagic (EHEC) and enteropathogenic *Escherichia coli* (EPEC) (3). They can all cause so-called “attaching and effacing lesions” in the intestinal mucosa – structural changes that lead to the destruction of microvilli and thus to a massive impairment of the intestinal barrier. This can lead to severe symptoms such as diarrhea and dehydration; in the case of EHEC, it can even lead to life-threatening complications such as hemolytic uremic syndrome (HUS) with renal failure (3).

The immune response to *C. rodentium* infection is complex and proceeds in several phases. In the early phase, innate immune cells in the colon – including macrophages, innate lymphoid cells (ILC) 3 and dendritic cells (DC) – are activated (4–6). Macrophages and DC´s take up bacterial antigens and present them, thereby initiating the adaptive immune response. Dendritic cells migrate via the lymphatic vessels to the draining mesenteric lymph nodes (mLN), where they activate both T and B cells and initiate antigen-specific differentiation (reviewed by (1)).

Th17 cells in the *lamina propria* play a central role in the immune defense. They control the infection by producing Interleukin (IL) -17 (7). As the infection progresses, B cells are also activated and differentiated. This results in an isotype switch; in which plasma cells produce antigen-specific immunoglobulins such as IgG1 and IgA which are crucial for the complete elimination of the pathogens (8). The activated B and T cells then return from the mLN to the inflamed tissue of the intestine.

Interestingly, there is an alternative model for B cell activation: In this scenario, activation and differentiation occur directly in the gut, for example in lymphoid structures such as Peyer’s patches, cecal and colonic patches, and isolated lymphoid follicles. Even when the mesenteric lymph nodes are removed, a pronounced, antigen-specific B cell response can still be observed in the small intestinal mucosa.

It has long been recognized that intestinal lymphatic drainage is segmentally organized. Esterházy and colleagues mapped this architecture in detail, showing that individual lymph nodes drain distinct and non−overlapping regions of the intestine (9). Despite this complex network, the specific contribution of individual lymph node compartments to adaptive immunity against intestinal pathogens remains incompletely understood. This question is particularly relevant for infections such as *C. rodentium*, where the immune response integrates cues from multiple gut regions and lymphoid tissues, yet the dominant effector phase ultimately occurs in the colon. Whether lymph nodes draining the colon are required for generating effective adaptive immunity, or whether colonic immune responses can be maintained by alternative lymphoid structures when these nodes are absent, remains a central open question. Building on this, we now show that these regional lymph nodes play a specific role in the immune response against *C. rodentium* – and that the absence of individual lymph nodes cannot be compensated for by others. For this purpose, either the colon−draining lymph nodes or all mesenteric lymph nodes were removed, and the resulting effects on colonic Th17 effector responses and plasma cell differentiation were assessed.

## Materials and methods

### Mice

Female C57BL/6J (B6J) were bred at the Central Animal Facility of the Hannover Medical School and were used at the age of 8–10 weeks. Mice were housed in a room with a controlled environment and 12 hour light/dark cycle. If not stated otherwise, mice received pelleted diet (Altromin 1324 TPF, Altromin Spezialfutter GmbH & Co. KG, Lage, Germany) and autoclaved water ad libitum. Cages were lined with softwood granulate (poplar wood, ANT-Tierhaltungsbedarf, Buxtehude, Germany). The mice received Sizzle Nest paper material, a cotton nesting pad and a mouse house (all ANT-Tierhaltungsbedarf). Animals were monitored according to FELASA recommendations (10) and did not reveal any evidence of infection with common murine pathogens except for *Helicobacter* sp., *Klebsiella oxytoca*, *Rodentibacter* sp, *Staphylococcus aureus*, *Chilomastix* sp., *Trichomonas* sp.

### Ethics statement

This study was conducted in accordance with German animal protection law and with the European Directive 2010/63/EU. All experiments were approved by the Local Institutional Animal Care and Research Advisory committee (Hannover Medical School) and permitted by the Lower Saxony State Office for Consumer Protection and Food Safety (LAVES; file number: 20/3445).

### Experimental setup

**Figure 1:**
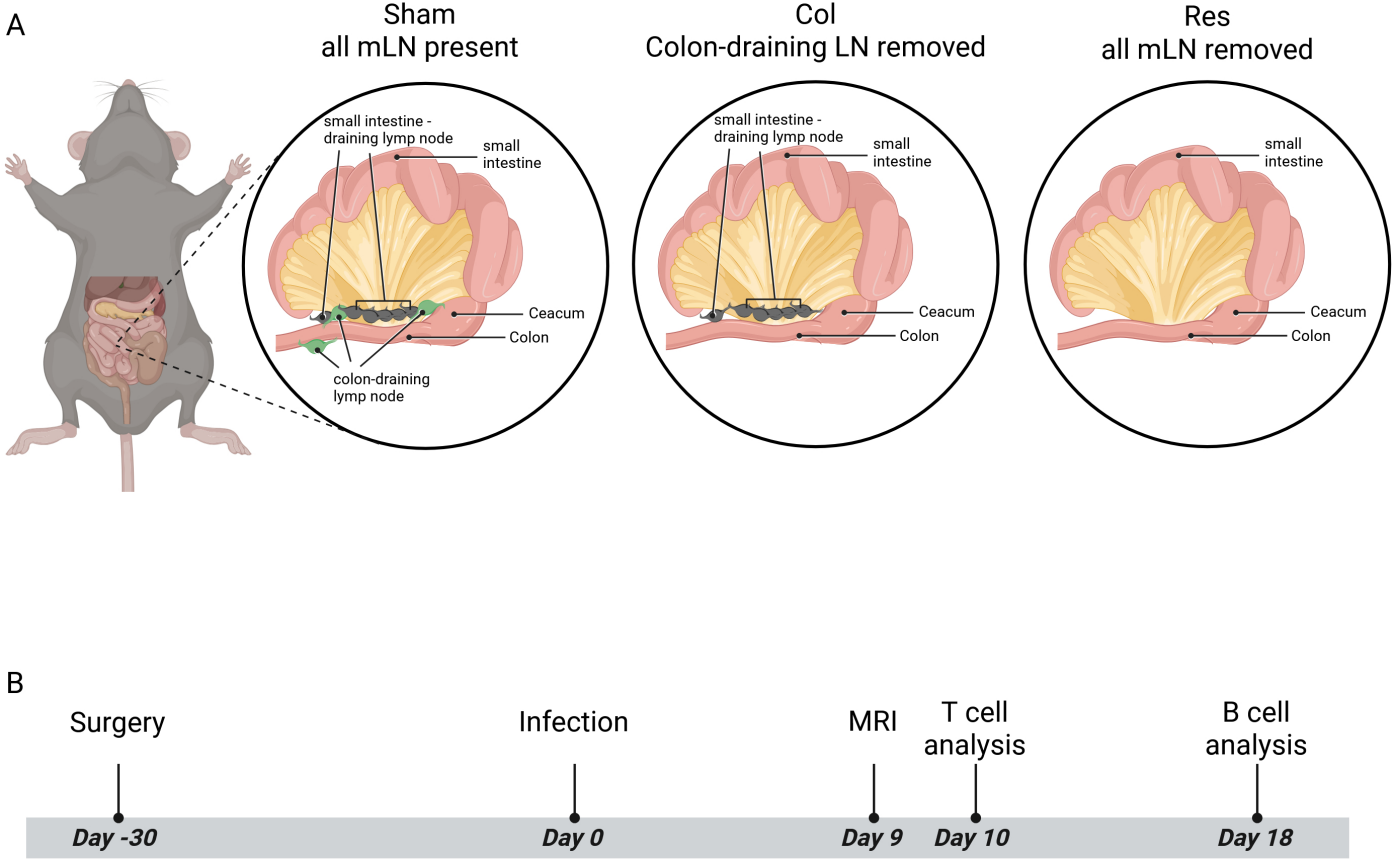
Schematic representation of intestinal lymph node anatomy and experimental workflow. **(A)** Schematic illustration of a mouse showing the anatomical location of the intestinal tract and the associated lymphatic drainage network. The magnified panel highlights the major gut−draining lymph nodes, including small−intestine–draining nodes and colon−draining mesenteric lymph nodes, as well as the relative positions of the small intestine, cecum, and colon. This schematic provides anatomical context for the lymph node resection procedures performed in the study. Created in BioRender. Basic, (M) (2026) https://BioRender.com/cnqvwsz **(B)** Overview of the experimental timeline. Mice underwent surgery 30 days before infection, followed by oral inoculation with *Citrobacter rodentium* on day 0. MRI analysis was performed on day 9, T cell analyses on day 10, and B cell analyses on day 18 post−infection.

The study involved performing intestinal surgery on all experimental animals (**Figure 1**), followed by a standardized four−week recovery period. To visualize the anatomical context relevant to the resection procedures, **Figure 1A** provides a schematic representation of the intestinal lymphatic drainage network, highlighting the major gut−draining lymph nodes. A schematic overview of the experimental workflow, including infection timeline, and cell analysis, is provided in **Figure 1B**.

C57BL/6J mice were used in this study because they represent the standard background for *C. rodentium* infection models and allow for well−characterized, reproducible immune responses without severe disease. To induce acute colitis at a defined time point, all operated animals were orally infected with *C. rodentium* ICC180 after completion of the recovery phase. Depending on the immune cell population of interest, animals were euthanized either on day 10 post−infection for T−cell–focused analyses or on day 18 post−infection for B−cell–associated readouts (7, 8). In the T−cell cohort, animals additionally underwent *in vivo* MRI imaging on day 9 post−infection to assess intestinal inflammation. All samples were collected post−mortem during necropsy for subsequent flow cytometric, molecular, immunological, and histopathological analyses. The experiments were conducted in 20 independent trials. Across trial runs, tissues were collected for histological scoring, flow cytometry, and real−time PCR. Serum samples were obtained from all animals, and fecal samples were collected from one animal per cage to determine bacterial load (CFU/g).

### Intestinal surgery

As described earlier (11, 12), all mLN of the small and large intestine or only colon-draining mLN (cmLN) of mice were removed. Mice were anesthetized with combined anesthesia of ketamine (Anesketin^®^ 100 mg/mL; 100 mg/kg), xylazine (Rompun^®^ 20 mg/kg; 2.8 mg/kg KGW) and Midazolam (0.7 mg/kg). Before initiation of anesthesia, animals received atropine (Atropinsulfat 0.5 mg/mL, 0.05 mg/kg KGW) and meloxicam (Metacam^®^, 2 mg/mL, 1 mg/kg KGW) subcutaneously. During the procedure and postoperatively, the animals were placed on a heated blanket which were covered with strile cloth. The corneas were protected from drying out during anesthesia using eye ointment (Bepanthen^®^; Bayer, Leverkusen, Germany). After ensuring the depth of anesthesia by checking the inter-toe reflex, the animals’ abdomens were shaved and disinfected with Braunol^®^ (Braun, Melsungen, Germany). The skin, abdominal muscles and peritoneum were opened with an approx. 1.5 cm long incision along the linea alba. During the intervention, the animals were randomly assigned to the different groups (Res, Col, Sham) and received the corresponding operations. Depending on which intervention the animal received, either all lymph nodes were resected (Res), only the colon draining lymph nodes (Col) or no lymph nodes (Sham) were resected. The intestine was then repositioned and the abdominal cavity closed by peritoneal suture after i.p. administration of sterile PBS, for which an absorbable VICRYL™ suture was used (Ethicon^®^). The skin was closed by applying a staple suture with staples for veterinary use (Autoclip^®^Physicians Kit). Animals were under constant observation on warming blankets until they were fully awakened from anesthesia.

For post-operative analgesia, mice received daily meloxicam (Metacam^®^, 2 mg/mL, 1 mg/kg KGW) subcutaneously during the first three days after surgery. The animals had a regeneration period of four weeks after the operation, during which time pseudoafferent lymph vessels formed (12, 13).

### Infection model

To induce acute colitis at a fixed time point, the animals were infected with *Citrobacter rodentium* ICC180 after a recovery period of 4 weeks. *C. rodentium* ICC180 (14) was kindly provided by M. Lochner. Bacteria from the glycerol stock were added to LB agar containing nalidixic acid sodium salt (NASS), an inhibitor of bacterial polymerase, using a three-loop smear without thawing the glycerol stock. NASS was used in the experiment because of the *C. rodentium* used is resistant to this substance and the agar therefore has a slight selectivity for this pathogen. The inoculated agar plate was incubated overnight at 37 °C. On the following day, a single colony was picked using a flamed smear loop and transferred into an Erlenmeyer flask containing 10 mL sterile LB medium (Carl Roth GmbH + Co. KG, Karlsruhe DE). The culture was incubated unsealed for 12 hours in a shaking incubator (37 °C, 180 rpm). After 12 hours of incubation, a further 50 mL of LB medium was added to the culture. Following a further 30 min of shaking incubation, the optical density at 600nm (OD600) was measured for the first time using a photometer (Eppendorf AG, Hamburg DE). Animals were infected by oral gavage with 1 x 10^8^ to 1 x 10^9^ CFU in 100 µL PBS. Control animals received the same amount of PBS. Depending on the target cell population to be analyzed animals were euthanized at day 10 post infection for T cell analysis or at day 18 post infection for B cell analysis.

### MRI performance

After an infection phase of 9 days, the test animals of the T cell study were submitted to imaging. A Bruker 7T Pharmascan 70/16 (Bruker Corporation, Ettlingen Germany) was used to generate magnetic resonance images of each individual animal. To inhibit intestinal motility, animals were administered a solution of clonidine hydrochloride (Sigma Life Science, St. Louis) dissolved in sterile isotonic sodium chloride solution at a concentration of 10 μg/kg for veterinary use (B. Braun Melsungen AG) s.c. at 0.1mL/10g body weight prior to anesthesia for magnetic resonance imaging (MRI). Anesthesia was induced with isoflurane (3% isoflurane in oxygen) and the animals were then taken directly to the tomograph. During the MRI, the animals received a maintenance dose of approx. 1-2% isoflurane in oxygen via a non-invasive breathing mask. With the help of a respiratory monitor (SAII Model 1030 Monitor, Stony Brook, NY, USA), the isoflurane concentration was titrated so that an optimal respiratory rate could be achieved. The maintenance of body temperature during anesthesia was ensured by positioning on a temperature control pad with warm water circulation. At a field strength of 7 T and a shielded gradient system (300 mT/m), individual sectional images were obtained. The repetition time was TR = 2266.9 ms with echo times (TE) of 11, 22 and 33 ms. The animals were kept under constant observation until the anesthesia had completely worn off, after which they were returned to their original cages and thus to the animal room.

### MRI evaluation

The MRI slice images were preprocessed by the vendor software (Bruker Paravision 6.01) and exported by the MHH Small Animal Imaging Center and made available for evaluation. Dicon files with 44 image segments at the above-mentioned echo times were available for each test animal. The Echo Time of 22ms was used for evaluation. Sagittal sections of the abdomen were evaluated in the T2 weighting, as this is the ideal place to interpret the soft tissue structures. To determine the degree of inflammation, the wall thickness of the rectum and colon was measured using the image processing software ImageJ (open source). For this purpose, 3 images were selected for each bowel section, which show a clear cross-section of the colon/rectum. The image sequence of the rectum was defined as the most aborally lying contiguous images, which showed a clear section through the rectum. For the colon image sequence, the 3 images directly adjacent to the caecum were used. The inner and outer walls were then measured clockwise at 8 points each using the ImageJ measurement tool, resulting in an average wall thickness. After transfer to a table with the distances and the corresponding angles, the values were uploaded to https://calliope.shinyapps.io/mrtsum/and automatically averaged there.

### Determination of the bacterial load

To determine the bacterial load of the infected mice and to confirm the absence of infection in control animals, fecal samples were collected from the animals. Each sample was weighed and subsequently homogenized in sterile PBS using sterile plungers. Serial dilutions ranging from 1x10–^1^ to 1x10–^8^ were prepared and plated onto selective NASS LB agar plates. After 24 hours of incubation, the CFUs were counted. For analysis, the highest dilution yielding countable CFUs was used. Based on the CFU counts and the corresponding sample weight, bacterial loads were calculated and expressed as CFU per gram of feces (CFU/g).

### Histology

Colon samples were prepared as modified “Swiss roll”, fixed in neutral buffered 4% formalin, embedded in formalin, sectioned at 5-6 µm and stained with hematoxylin and eosin.

The histological preparations of the *C. rodentium* infected animals were randomly examined under a light microscope for epithelial hyperplasia (the score marks the degree of hyperplasia compared to the control) and cellular infiltration with mononuclear cells (0 = no infiltration, 1 = slight infiltration, 2 = moderate infiltration, 3 = strong infiltration). The scores are summed up to a maximum value of 6 (**Table 1**) (14). Sample evaluation was performed in a blinded fashion, and scoring was carried out independently by two investigators who were unaware of the group allocation. Images were taken with a camera light microscope (Zeiss, Axio Imager, Axiocam 305 color) and the Zeiss ZEN light Microscope software.

**Table 1:** The acute colitis histology scoring of *C. rodentium* model.

Epithelial damage and hyperplasia	Score	Cellular infiltration of mononuclear cells	Score
Normal crypt structure, normal crypt length, no epithelial damage	0	No mononuclear cells in lamina propria (or rare single cells)	0-0,5
mild crypt hyperplasia (locally confined)	0,5-1,0	Locally confined infiltration (submucosa not affected)	1,0-1,5
Mild Hyperplasia (affects larger area)	1,0-1,5	Locally confined infiltration (submucosa affected)	2
Hyperplasia (locally confined/larger area)	2,0-2,5	Infiltration of larger area, submucosa affected, occasionally infiltration between crypts	2,5
Hyperplasia (pronounced, affects larger area)	2,5-3,0	Infiltration of larger area, pronounced infiltration, submucosa affected, pronounced infiltration between crypts	2,5-3,0

### Statistical analysis

Statistical analyses were performed using GraphPad Prism^®^ 10. Sample sizes were determined by power analysis using G*Power. Prior to statistical testing, all datasets were examined for normal distribution using the Shapiro–Wilk test. Outliers were identified using Grubbs’ test for normally distributed data or the ROUT method for non−parametric datasets and were removed only when justified by test criteria. Depending on data distribution, comparisons between experimental groups (Sham, Res, Col) and PBS−treated controls were performed using one−way ANOVA with Šidák or Holm–Šidák post−hoc correction, or the Kruskal–Wallis test with Dunn’s post−hoc test for non−parametric data. All tests were two−tailed, and statistical significance was defined as P < 0.05. Data are presented as individual values with mean ± 95% confidence intervals unless otherwise indicated.

### Magnetic cytokine assays

The cytokine levels of BAFF, IFNg, IL6, IL17A, IL33, IL10, IL17E/IL25 and TNFa were measured in serum (diluted 1:2) using a mouse premixed Luminex assays (Bio-techne, Minneapolis, USA). The assay was performed according to the manufacturer’s instructions. The following cytokines were below the detection limit IFNg, IL6, IL33, IL17E/IL25 and TNFa.

### Measurement of serum lipocalin-2 by ELISA

Serum samples were collected post-mortem during necropsy and stored at −20 °C until analysis. Lipocalin-2 concentrations were determined using the Mouse Lipocalin-2 DuoSet^®^ ELISA (R&D Systems), according to the manufacturer’s instructions. Samples were diluted 1:1000 in the recommended diluent. Optical density was measured at 450 nm with reference correction at 540 nm using a microplate spectrophotometer (Varioskan™ LUX, Thermo Fisher Scientific). Concentrations were calculated based on a standard curve generated by four-parameter logistic (4PL) regression using SkanIt software (Thermo Fisher Scientific).

### Detection of C. rodentium specific Immunoglobulin Isotypes by ELISA

To quantify *C. rodentium* specific immunoglobulin isotypes, the Mouse Uncoated ELISA Ig Isotyping Kit (Invitrogen, Thermo Fisher Scientific) was used. Serum samples were collected post-mortem during necropsy and stored at −20 °C until analysis. Samples were diluted 1:1000 in the recommended buffer. Heat-inactivated *C. rodentium* lysate was used as the capture antigen. The ELISA was performed according to the manufacturer’s instructions using isotype-specific detection antibodies included in the kit (IgG1, IgG2a, IgA; all diluted 1:250). Optical density was measured at 450 nm with reference correction at 540 nm using a microplate spectrophotometer (Varioskan™ LUX, Thermo Fisher Scientific). Isotype-specific antibody levels were quantified and expressed relative to the control group.

### Isolation of immune cells from colon

For isolation of colonic immune cells, the colon was freed of fat and flushed with cold DPBS. Then, it was cut open longitudinally and cut into three to four pieces. Afterwards, colon was incubated in buffer 1 (HBSS, 0.5 M EDTA, 0.9% FCS) for 15 minutes at 37 °C and 220 rpm. After vortexing, the supernatant containing the loosened cells was collected in another 50 mL tube and stored on ice. Lastly, 10 mL of buffer 2 (RPMI1640, FCS, Collagenase A (125U/mg), DNase (5mg/mL) was added and incubated under the same condition but for a total of 20 minutes. The supernatant was collected as described for buffer 1 and added to the cell suspension. Cells were spun down for 10 min at 500 x g and 4 °C. To ensure a cleaner cell population a Percoll-gradient was performed. Cells were then stained directly for flow cytometry.

### Flow cytometry staining

#### Extracellular staining

For cell staining, 1 x 10^6^ cells were transferred into a 96-well U-bottom plate. Per staining 50 µL of antibody dilution was added. All antibodies used for extracellular staining (**Table 2**) were diluted in MACS buffer. Unstained control cells were suspended in 50 µL of MACS buffer. Staining was performed for 20 minutes at 4 °C. Finally, cells were suspended in 250 µl MACS buffer and were ready to be analyzed by flow cytometry (for gating strategy, see appendix).

**Table 2:** Antibodies used for extracellular staining for flow cytometry.

Antigen	Fluorophore	Clone	Dilution	Manufacturer
CD138	PE	281-2	1:500	BioLegend^®^
CD185, CXCR5	BV421	L138D7	1:200	BioLegend^®^
CD19	BV421	6D5	1:500	BioLegend^®^
CD19	FITC	1D3	1:1000	BioLegend^®^
CD25	APC	PC61	1:1000	BioLegend^®^
CD279, PD-1	PE/Dazzle	29F.1A12	1:200	BioLegend^®^
CD3	FITC	17A2	1:250	BioLegend^®^
CD38	APC/Cy7	90	1:1000	BioLegend^®^
CD4	PE-Cy7	RM4-5	1:2000	BioLegend^®^
CD44	AF647	IM7	1:2500	BioLegend^®^
CD45R/B220	VioBlue	RA3-6B2	1:500	MACS
CD69	PerCP/Cy5.5	H1.2F3	1:200	BioLegend^®^
CD8a	APC/Cy7	53-6.7	1:1000	BioLegend^®^
CD90.2	APC Cy7	30-H12	1:2000	BioLegend^®^
CD98	PE/Cy7	RL388	1:1000	BioLegend^®^
GL7 Antigen (T/B Cell Act.)	PE/Cy7	GL7	1:200	BioLegend^®^
IgA	APC	mA-6E1	1:200 (1:500)	Invitrogen
PNA	FITC		1:1000	Vector Laboratories
Sca1	APC/Cy7	D7	1:500	BioLegend^®^

#### Intracellular staining

Intracellular staining was performed after extracellular staining. Cells were treated with the True-Nuclear™ Transcription Factor Buffer Set (BioLegend^®^, California, USA) according to the manufactures protocol and stained with antibodies listed in **Table 3**.

**Table 3:** Antibodies used for intracellular staining for flow cytometry.

Antigen	Fluorophore	Clone	Dilution	Manufacturer
IL10	PE	JES5-16E3	1:500	Biolegend, USA
IL17a	AF700	TC11-18H10.1	1:500	Biolegend, USA
IFN-y	PE	MF-14	1:500	Biolegend, USA

### Quantitative real time PCR

#### RNA isolation and cDNA synthesis

For the isolation of RNA from animal tissue the RNA isolation kit (RNeasy Mini Kit, Qiagen) was used according to the manufacturer’s recommendations. RNA measurement was performed with a NanoDrop (Spectrometer ND-1000, Peqlab Biotechnologie GmbH).

The QuantiTect Reverse Transcription Kit (Qiagen) was used to synthesize complementary DNA (cDNA) according to the manufacturer’s protocol.

#### qPCR setup

TaqMan^®^−based quantitative real−time PCR was performed using TaqMan^®^ Fast Advanced Master Mix (Thermo Fisher Scientific) and assay primers/probes listed in **Table 4**. Reactions were run on a QuantStudio™ Flex Real−Time PCR System (Applied Biosystems). Gene expression was quantified using β−actin (Actb) as the endogenous control. Ultrapure HPLC−grade water served as the no−template control. All reactions were prepared and analyzed in technical triplicates on MicroAmp^®^ Optical 384−Well Reaction Plates (Thermo Fisher Scientific).

**Table 4:** Primer list: overview of primers used for gene expression analysis.

Gene	Gene name	Primer-assay-ID
*Actb*	beta-Actin	Mm00607939_s1
*Il21*	Interleukin 21	Mm00517640_m1
*Ighg1*	IgG1	Mm05833521_m1
*Igip*	IgA producing Protein	Mm04336752_s1

This overview shows the primers used for the TaqMan^®^ fast advanced master mix assay.

PCR amplification was carried out under the following cycling conditions:

50 °C for 2 min,
92 °C for 10 min,
followed by 40 cycles of
95 °C for 1 s (denaturation) and
60 °C for 20 s (annealing/extension).

Data were analyzed using Thermo Fisher Connect™ software, and relative transcript abundance was calculated using the 2^-^ΔCt method. The resulting RQ values were subsequently exported to GraphPad Prism 10 for statistical analysis and visualization.

## Results

### T cell responses at an early post−infection stage are unaffected by mLN resection, accompanied by small differences in B cell–related readouts

**Figure 2:**
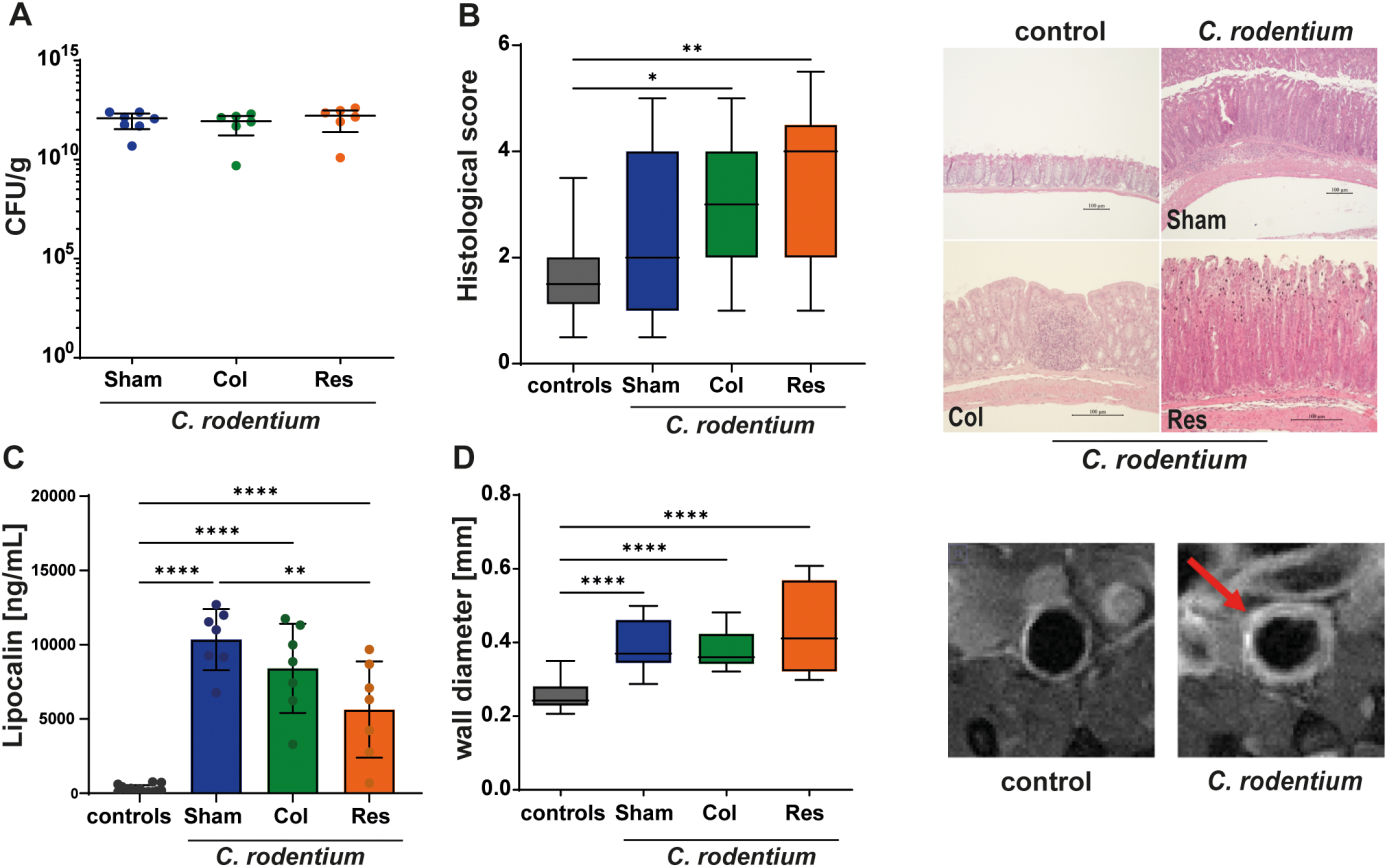
Early intestinal inflammation following *C. rodentium* infection in mice with differential mesenteric lymph node resection. Ten days after inoculation with *C. rodentium*, bacterial burden in stool **(A)**, histological inflammation of the colon **(B)**, and serum lipocalin−2 concentrations **(C)** were assessed. Control animals received PBS. The analysis included three surgical groups: Sham (no mLN removed), Res (complete mLN resection), and Col (resection of the colon−draining mLN). MRI was performed on day 9 post−inoculation to determine colonic wall thickness **(D)**. **(A)** *C. rodentium* was detectable in stool samples of all infected groups, independent of the surgical procedure. CFU/g values are presented as scatter plots with mean ± 95% CI (n = 6–7). **(B)** Histological scoring revealed robust inflammation in all infected groups, with the Col and Res groups showing the strongest pathology compared with PBS−treated controls. Scores are shown as box−and−whisker plots indicating median, minimum, and maximum values (n = 11–28). Representative hematoxylin− and eosin−stained colon sections from infected mice are included. **(C)** Serum lipocalin−2 levels were markedly elevated in infected animals relative to controls at day 10 post−inoculation. Values (ng/mL) are displayed as scatter plots with bars showing mean ± 95% CI (n = 7–14). **(D)** MRI performed on day 9 post−inoculation demonstrated a pronounced increase in colonic wall thickness in all infected groups (Sham, Res, and Col) compared with controls. Scatter plots with bars show mean ± 95% CI. Representative MRI images from infected and control mice are shown. Statistical analyses were performed using ANOVA with Šidák post−hoc testing or the Kruskal–Wallis test with Dunn’s post−hoc test. Significant differences are indicated by P < 0.05 (*), P < 0.01 (**), and P < 0.0001 (****).

As *C. rodentium* is a well-known pathogen that induces specific Th17 responses with increased levels of IL17 (15), we focused on T cell induction in the colon of mice subjected to different mesenteric lymph node (mLN) resection procedures (**Figure 1**). Four weeks after surgery—during which mice either underwent a sham procedure (Sham), complete removal of the mLN (Res), or selective resection of the colon−draining node (Col), animals were orally infected once with 1 × 10^9^ CFU *C. rodentium* for 10 days. *C. rodentium* was similarly detected in feces of all infected groups and to marked inflammation as determined by histological evaluation and MRI (**Figures 2A–D**). Hyperplasia and infiltration of mononuclear cells were observed in the colons of these animals. An increased colon wall thickness was observed in the MRI. Furthermore, all groups showed increased lipocalin 2 levels in the serum, whereas non infected animals had low lipocalin levels, as expected (**Figure 2C**).

**Figure 3:**
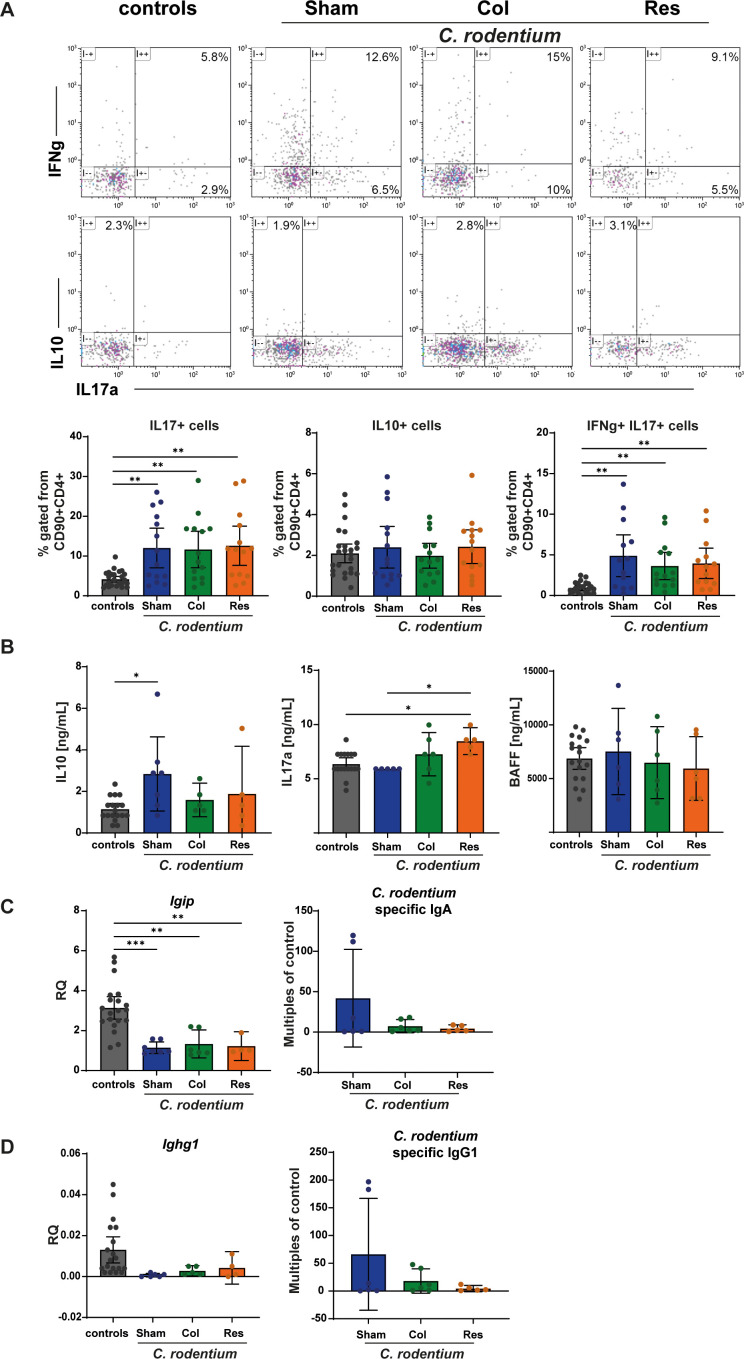
Cellular, cytokine, and antibody responses 10 days after *C. rodentium* inoculation. Ten days after inoculation with *C. rodentium*, flow cytometric analysis of colonic lymphocytes **(A)**, serum cytokine profiling **(B)**, real−time PCR of colonic tissue **(C, D)**, and quantification of *C. rodentium*−specific serum immunoglobulins **(C, D)** were performed. Control animals received PBS. Analyses included all three surgical groups: Sham (no mLN removed), Res (complete mLN resection), and Col (resection of the colon−draining mLN). Gating strategies are shown in **Supplementary Figures 1**, **2**. **(A)** Colonic lymphocytes were assessed for Th17 (CD90^+^CD4^+^IL-17^+^), IL−10–producing CD90^+^CD4^+^ T cells, and IFNγ^+^IL−17^+^ T cell populations. All infected groups showed increased frequencies of Th17 cells relative to PBS controls. Data are displayed as scatter plots with mean ± 95% CI (n = 13–31). **(B)** Serum concentrations of IL−10, IL−17A, and BAFF were quantified by Luminex ELISA. Cytokine levels (ng/mL) are presented as scatter plots with bars indicating mean ± 95% CI (n = 5–18). **(C, D)** Real−time PCR revealed higher expression of Igip and Ighg1 in PBS−treated controls compared with infected mice. Values are shown as scatter plots with mean ± 95% CI (n = 4–20). Serum immunoglobulins specific for **(C)** rodentium antigens were measured by ELISA; Sham animals displayed higher levels of antigen−specific IgA and IgG1 relative to Res and Col groups. Data represent fold change relative to controls and are shown as scatter plots with mean ± 95% CI (n = 5–6). Statistical analyses were performed using ANOVA with Šidák post−hoc testing or the Kruskal–Wallis test with Dunn’s post−hoc test. Significant differences are indicated as P < 0.05 (*), P < 0.01 (**), P < 0.001 (***).

Using flow cytometry, we analyzed IL-17 production within the CD90^+^CD4^+^ lymphocyte compartment in the colon, which encompasses Th17-like effector cells activated during *C. rodentium* infection, independent of their precise cellular origin. As expected, we detected a pronounced IL-17–producing CD90^+^CD4^+^ cell population in all infected animals (**Figure 3A**), including both classical Th17-like and IFNγ^+^IL-17^+^ subsets. Importantly, the accumulation and cytokine production of these cells occurred independently of the presence of the mesenteric lymph nodes. In contrast, we were able to detect increased levels of IL17a in the serum of mLN resected infected animals. Whereas the mLN-bearing animals had increased levels of IL10 (**Figure 3B**). IL-10 production was used as a functional marker of immunoregulatory activity and does not allow discrimination between FoxP3^+^ regulatory T cells and other IL-10–producing CD4^+^ T cell subsets. Furthermore, we measured decreased expression of *Igip* (IgA inducing protein) and *Ighg1* (Immunoglobulin Heavy Constant Gamma 1) in all infected mice. In addition, we detected low concentrations of *C. rodentium* specific antibodies in the serum of infected animals (**Figures 3C, D**).

In summary, our data demonstrate that mLN are dispensable for local T cell–mediated immune responses during *C. rodentium* infection, including the accumulation and IL−17 production of Th17 cells in the colon.

### mLN-dependent B cell activation and immunglobuline production in response to *C. rodentium*

**Figure 4:**
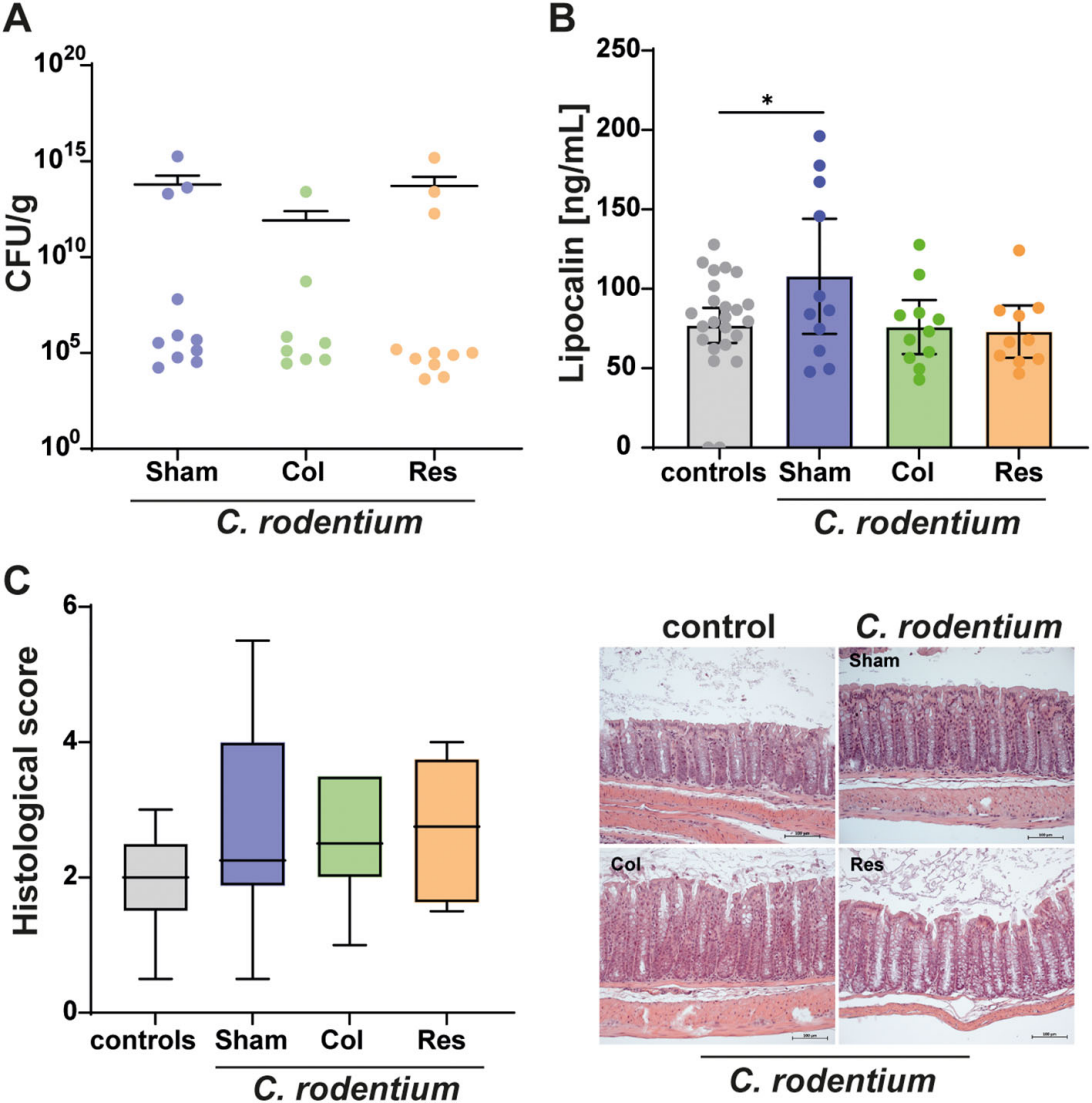
Colonic inflammation 18 days after *C. rodentium* inoculation. Eighteen days after inoculation with *C. rodentium*, bacterial burden in stool **(A)**, serum lipocalin−2 concentrations **(B)**, and histological inflammation of the colon **(C)** were assessed. PBS−treated animals served as controls and were shared across all experimental groups. Analyses included the three surgical cohorts: Sham (no mLN removed), Res (complete mLN resection), and Col (resection of the colon−draining mLN). **(A)** *C. rodentium* was detected in fecal samples of all infected groups. CFU/g values are presented as individual data points with mean ± 95% CI (n = 8–11). **(B)** Serum lipocalin−2 concentrations were elevated in all infected mice relative to PBS controls. Values (ng/mL) are shown as scatter plots with bars indicating mean ± 95% CI (n = 8–36). **(C)** Histological scoring revealed increased inflammation in all infected groups compared with controls, with representative hematoxylin− and eosin−stained colon sections included. Scores are displayed as box−and−whisker plots depicting median, minimum, and maximum values (n = 8–36). Statistical analyses were performed using ANOVA with Holm–Šidák post−hoc testing or the Kruskal–Wallis test with Dunn’s post−hoc test. Significant differences are indicated as P < 0.05 (*).

Next, the influence of the mesenteric lymph nodes on B cell differentiation during *C. rodentium* infection was examined. Animals were orally administered up to x 10^9^ CFU, and the B cell response in the colon was analyzed 18 days later. As in the earlier experiment, bacterial load in feces did not differ between the infected groups (**Figure 4A**). Likewise, no significant differences in histological inflammation scores or serum lipocalin−2 levels were detectable at day 18 (**Figure 4B**), indicating that tissue regeneration had begun in all infected animals. Mild crypt hyperplasia and mononuclear cell infiltration were observed in the infected groups but remained below significance and were absent in controls (**Figure 4C**).

**Figure 5:**
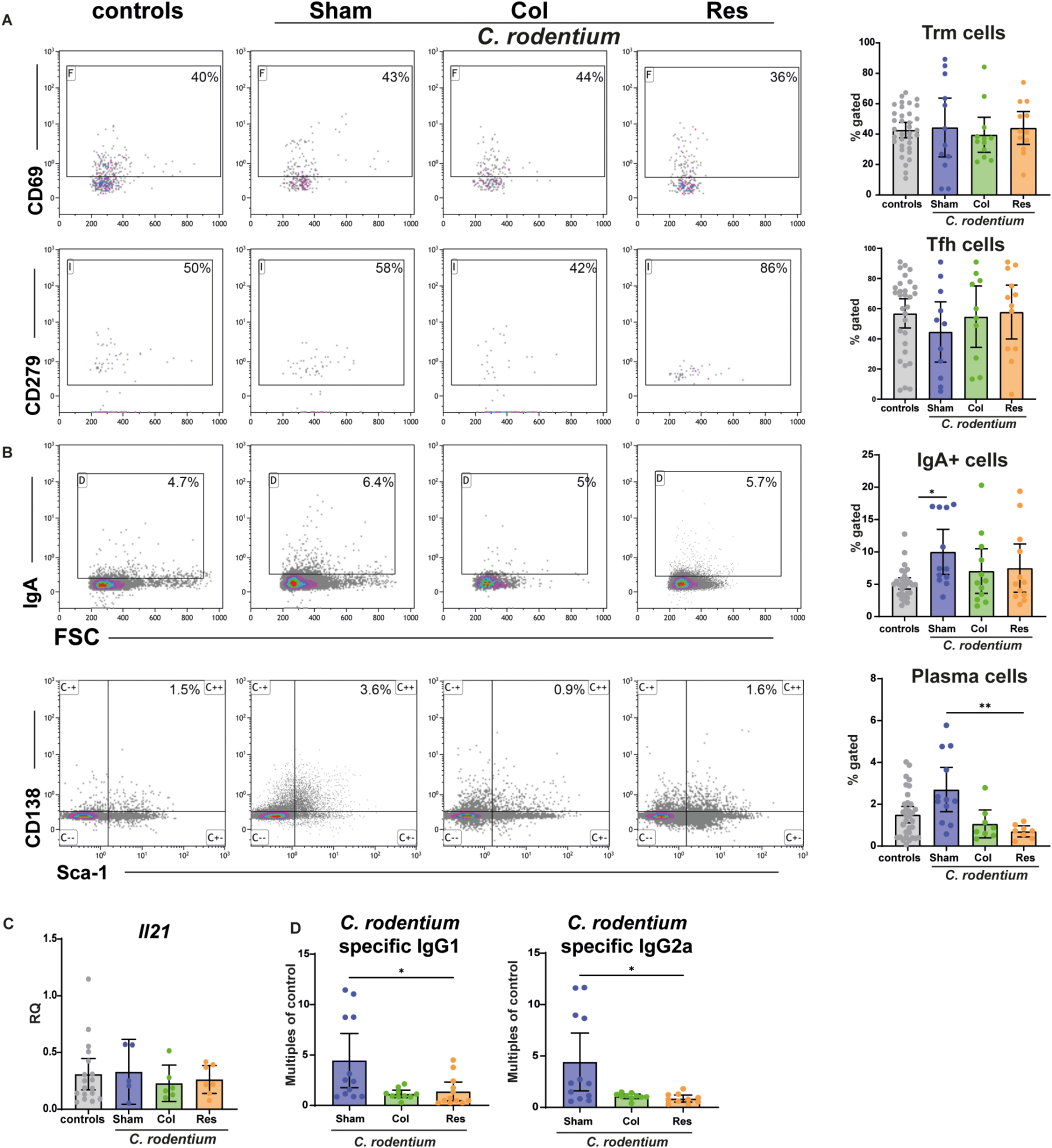
Cellular, transcriptional, and humoral responses 18 days after *C. rodentium* infection. Eighteen days after inoculation with *C. rodentium*, flow cytometric analysis of colonic lymphocytes **(A, B)**, real−time PCR of colonic tissue **(C)**, and serum ELISAs for *C. rodentium*−specific immunoglobulins **(C)** were performed. Analyses included all three surgical groups: Sham (no mLN removed), Res (complete mLN resection), and Col (resection of the colon−draining mLN). PBS−treated animals served as controls. **(A)** Colonic T lymphocytes were analyzed for Trm tissue−resident memory T cells; Tfh, T follicular helper cells, and *Il21* expression by real−time PCR. Data are presented as scatter plots with bars indicating mean ± 95% CI (n = 6–18). Gating strategies are shown in **Supplementary Figure 3**. **(B)** IgA^+^ B cells and plasma cells were quantified in colonic lymphocyte preparations. All infected groups exhibited detectable B cell activation, with Sham animals displaying higher frequencies of IgA^+^ B cells and plasma cells relative to the Res and Col groups. Data are shown as scatter plots with bars indicating mean ± 95% CI (n = 8–35). Gating strategies are provided in **Supplementary Figure 4**. **(D)** Serum immunoglobulins specific for *C. rodentium* antigens were measured by ELISA. Sham animals displayed increased antigen−specific IgG1 and IgG2a levels relative to Res and Col groups. Data represent fold changes relative to PBS controls and are presented as scatter plots with bars showing mean ± 95% CI (n = 10–12). Statistical analyses were performed using ANOVA with Holm–Šidák post−hoc testing or the Kruskal–Wallis test with Dunn’s post−hoc test. Significant differences are indicated as P < 0.05 (*) and P < 0.01 (**).

To further assess the adaptive immune response with regard to B cell differentiation, we first analyzed T cell populations known to support or reflect local humoral immunity. Tissue−resident memory T cells (Trm) were examined as indicators of prior local T cell activation, and CXCR5^+^PD−1^+^ T follicular helper–like (Tfh−like) cells were quantified because Tfh−mediated signals are essential for B cell maturation. No differences were observed between groups in either Trm or colonic Tfh−like cells at day 18 (**Figure 5A**), suggesting that local T cell activation states were largely preserved despite mLN resection.

We next analyzed colonic B cells. Animals retaining the full mesenteric lymph node chain (Sham) displayed higher frequencies of plasma cells and IgA^+^ B cells, whereas both mLN−resected groups (Res and Col) exhibited similarly reduced levels of these populations (**Figure 5B**). Because *C. rodentium* infection is restricted to the cecum and colon, B cell activation and class−switch recombination are expected to occur primarily within the colon−draining mLN. Thus, removal of either all mLN or only the colon−draining node disrupts the inductive sites required for generating IgA^+^ and plasma cell populations during colonic infection.

Consistent with this, *C. rodentium*–specific ELISAs showed increased antigen−specific IgG1 and IgG2a levels only in Sham animals, whereas both mLN−resected groups (Res and Col) failed to mount comparable systemic antibody responses (**Figure 5C**). Together, these findings demonstrate that, while local T cell activation in the colon is maintained, effective B cell differentiation and the development of systemic antigen−specific antibodies critically depend on the presence of the colon−draining mesenteric lymph nodes. Notably, lymph nodes draining the small intestine—still present in the Col group—could not compensate for the loss of colon−draining mLN, as Res and Col groups were indistinguishable across all analyzed parameters (**Figure 5**).

Taken together, these data demonstrate that antigen-specific B cell responses in the colon of mice are lymph node-dependent, whereas T cell-mediated inflammation occurs independently of lymph nodes. Furthermore, we showed that during colon inflammation, lymph nodes draining the small intestine do not take over the function of lymph nodes draining the colon.

## Discussion

Mesenteric lymph nodes (mLNs) are major immune structures in the abdomen. They drain lymph from the intestines and are essential for inducing immune responses or tolerance to intestinal antigens such as food proteins and commensal microbes. Their contribution to host defense is multifaceted: rather than functioning as a purely physical “firewall,” their protective capacity reflects immune filtering through phagocytosis of lymph−borne microbes as well as the generation of pathogen−specific IgG that prevents systemic pathogen dissemination. Observations from *Salmonella typhimurium* infection models illustrate this dual role, as removal of the mLN not only increases bacterial burden in peripheral organs but simultaneously eliminates the major site of pathogen−specific IgG production (16). Our findings align with this more nuanced interpretation.

In our study, we show that mLNs have a specific role in inducing a *C. rodentium* specific B cell response, but they are not required for generating a *C. rodentium* specific T cell response. This dichotomy aligns with the distinct anatomical and functional organization of the intestinal immune system. While dendritic−cell–driven T cell priming can occur locally within the colon or in alternative lymphoid tissues, B cell activation and class−switch recombination require the specialized microenvironment of the appropriate draining lymph node. Consistent with the non−invasive nature of *C. rodentium* (3), we did not detect systemic bacterial dissemination during infection. Markers of mucosal inflammation further supported this pattern: although histological and MRI−based alterations persisted at later time points, lipocalin−2 concentrations had already declined. Given its short half−life and dependence on neutrophil activity, lipocalin−2 frequently normalizes before structural tissue changes fully resolve and therefore provides an early indication of inflammatory resolution rather than a direct measure of residual epithelial damage.

The loss of mLNs is known to alter immune responses in both small and large intestine. Although some immune functions are maintained by other tissues, tolerance to commensals and inflammatory regulation are impaired (11, 16). Previous studies showed that in the absence of mLNs, IgA responses to oral antigens increase locally in the jejunum and ileum, while systemic IgM responses appear in the spleen (11).

Because different intestinal segments (duodenum, jejunum, ileum, colon) drain into distinct mLN regions, adaptive immune responses are spatially compartmentalized (9, 17). These lymph node compartments contain specialized stromal cells and immune cell compositions tailored to their specific microbial and dietary environments (9, 17). Although additional lymph nodes such as the iliac or caudal nodes may receive limited drainage from distal colonic regions, these structures are not considered primary inductive sites for colonic immune responses. Our finding that removal of colon−draining mLN alone phenocopied complete mLN resection argues against a compensatory role of these nodes in Th17 or B cell responses during C. rodentium infection. When individual lymph nodes are removed, neighboring nodes can sometimes compensate—for example, in the induction of oral tolerance—while other responses, such as against certain helminths, occur only in a single defined lymph node (9). In applying this concept to our system, only the colon−draining mLN are expected to contribute meaningfully to the immune response against a pathogen that is restricted to the cecum and colon. Our data reflect this principle: removal of colon−draining lymph nodes, or removal of the entire mesenteric lymph node chain, produced equivalent defects in plasma cell formation and antigen−specific antibody production.

Some mechanisms of *C. rodentium* specific immunity are already known. During infection, plasmacytoid dendritic cells (pDCs) migrate to the colon-draining lymph nodes and become more strongly activated than conventional DCs, upregulating pathogen-sensing receptors and inflammatory mediators (4, 18). pDC depletion enhances conventional DC activation, increases IFN-γ-producing Th1 cells, and reduces Treg and Th17 induction, resulting in excessive macrophage accumulation and heightened inflammation (4). These findings come from early infection stages; our data do not indicate a shift in the Th1/Th17 balance.

*C. rodentium* induces apoptosis in host cells and triggers a strong Th17 response (15, 19). We observed this Th17 response across all experimental groups, independent of mLN presence. During peak and late infection, IL-17 expression increases, and Th17 cells adopt a highly inflammatory, metabolically active profile (7). In addition, CD4^+^ tissue-resident memory T cells are induced independently of mLNs, as they do not express lymph-node-specific homing markers (20).

Another important T cell population during *C. rodentium* infection consists of CXCR5^+^PD-1^+^ T follicular helper (Tfh) cells, which develop in the mesenteric lymph nodes (mLN) and drive antigen-specific B cell responses (21). In Tfh-deficient mice infected with *C. rodentium*, reduced numbers of germinal center B cells and IgG1^+^ B cells were observed, and the IgG1 response could be restored by transferring Tfh cells (21). This activation and differentiation of B cells is essential for controlling *C. rodentium* at later stages of infection, as B cell-deficient mice fail to clear the pathogen (22). To assess T cell help within the tissue, we examined tissue−resident memory T cells (Trm) and CXCR5^+^PD−1^+^ T follicular helper–like (Tfh−like) cells in the colon itself. Both were preserved across all groups, indicating that local T cell activation in the colon does not require the mLN. Although these populations may contribute to cytokine support or localized immune tone, they cannot replace germinal center Tfh cells within the lymph node. Our findings reinforce that colonic tissue alone is insufficient to drive effective B cell class switching during infection.

In our study, we detected increased levels of *C. rodentium* specific antibodies and higher percentages of plasma cells in the colon after infection in mice that retained their mLNs. However, when the colon-draining lymph nodes -or all mLNs - were absent, these levels were markedly reduced. This highlights the importance of B cell activation and differentiation within the draining lymph nodes. Notably, we did not observe any differences in Tfh cell frequencies in the intestinal tissue itself, indicating that the defect arises specifically from the absence of the appropriate lymph node environment rather than from changes in intestinal Tfh cell populations.

Taken together, our findings demonstrate that acute local T cell–mediated immune responses during *C. rodentium* infection, including Th17 effector activity, do not require the draining lymph node and are sustained within the colon itself. In contrast, *C. rodentium*–specific B cell activation and differentiation critically depend on the mesenteric lymph nodes, as antigen−specific antibody levels are markedly reduced in their absence.

## Data Availability

The original contributions presented in the study are included in the article/**Supplementary Material**. Further inquiries can be directed to the corresponding author.
