# Supplementary material for: Mesenteric lymph nodes are required for B- but dispensable for local T cell effector responses following Citrobacter rodentium infection in mice
Source: Front Immunol. 2026 May 11;17:1812877. doi: 10.3389/fimmu.2026.1812877 (PMC13199288; doi:10.3389/fimmu.2026.1812877)
Supplement: Supplementary file 1 [file Table1.docx]

**Supplemental Figure**

Suppl. Fig.1


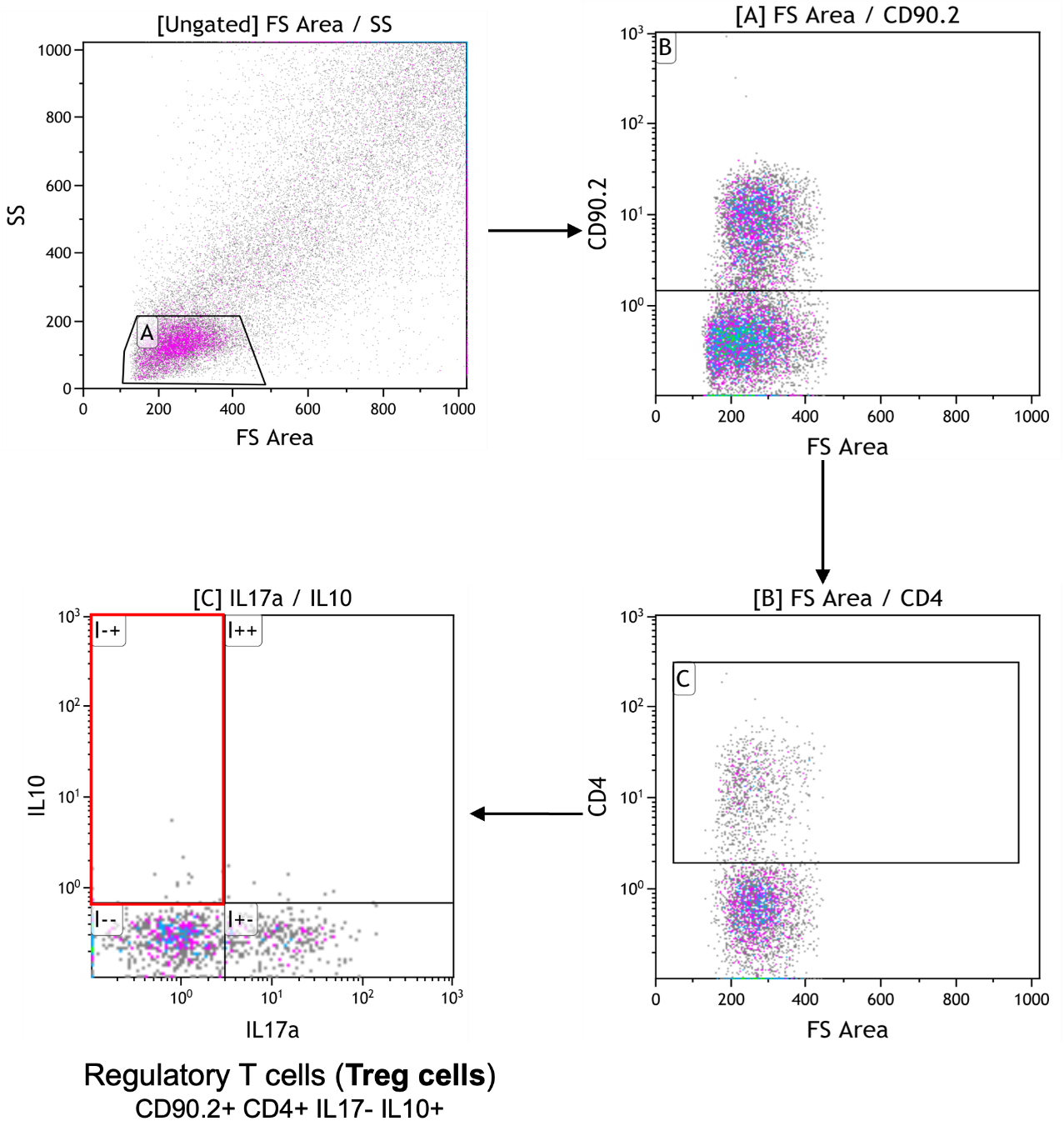


Suppl. Fig 1: Gating for IL10-producing CD90+CD4+ cells

Suppl. Fig.2


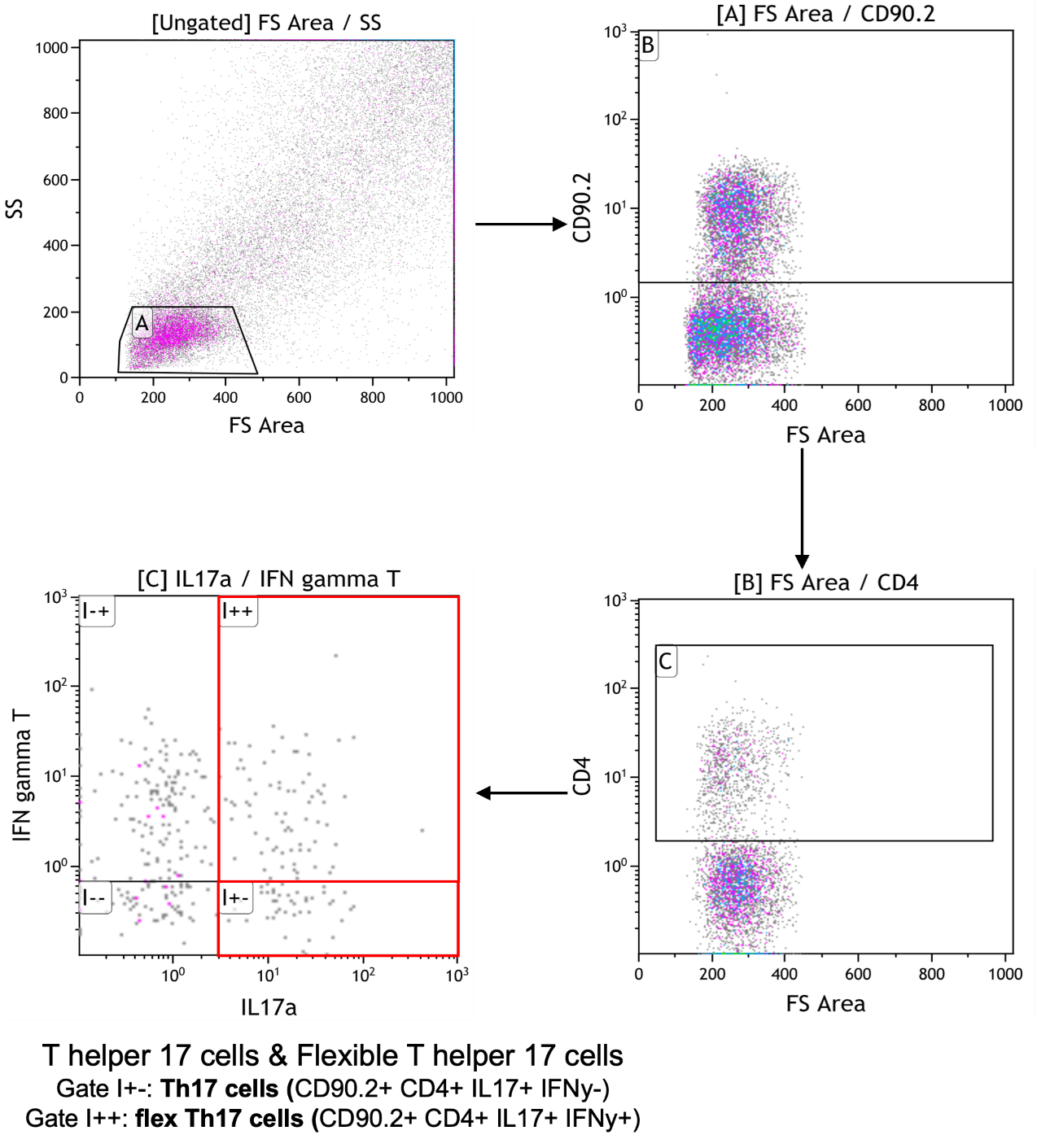


Suppl. Fig 2: Gating for Th17 (CD90⁺CD4⁺IL‑17⁺) and IFNγ⁺IL‑17⁺  CD4^+^CD90^+^ cell population

Suppl. Fig.3


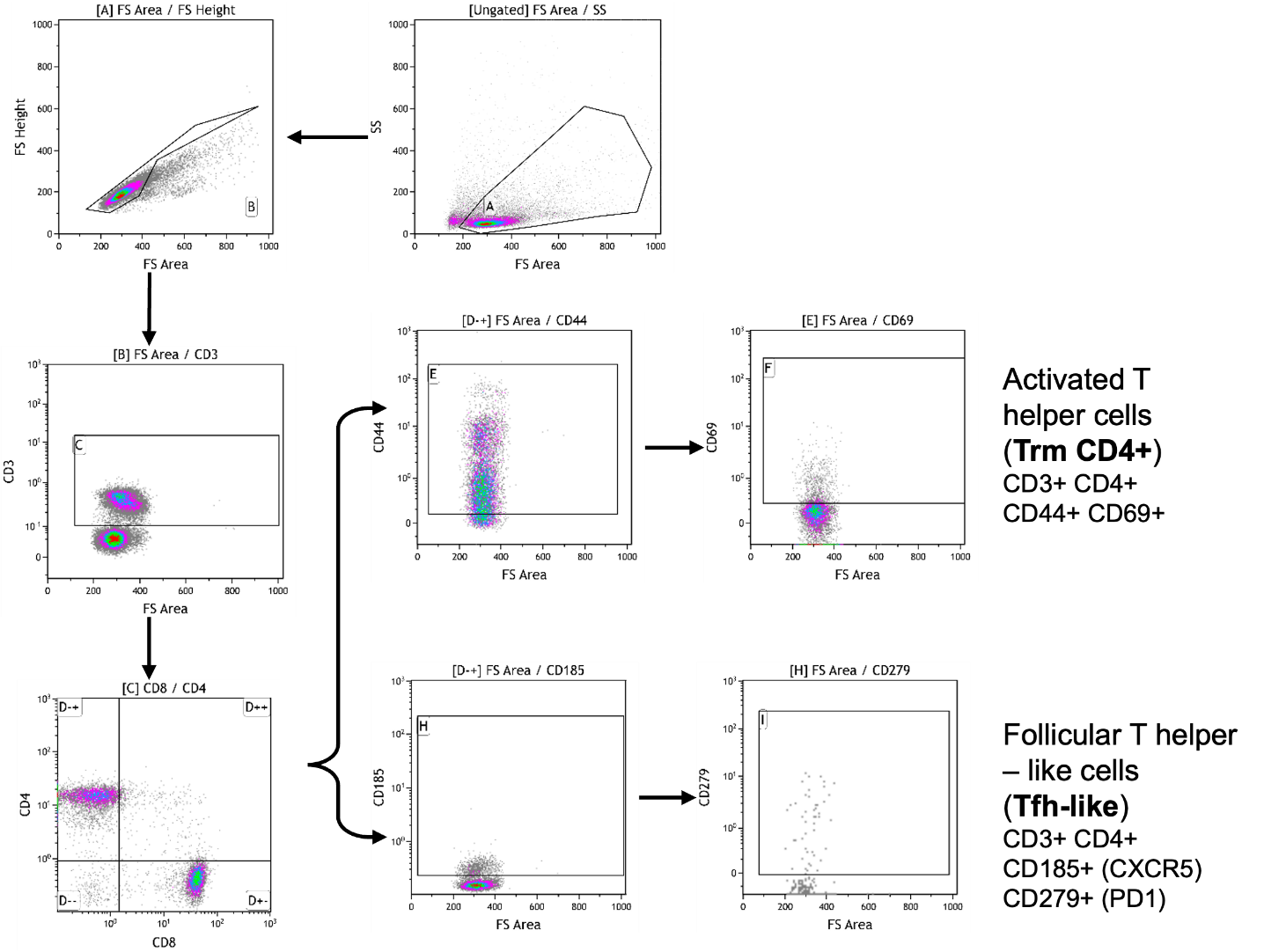


Suppl. Fig 3: Gating for activated T helper cells and follicular T helper cells

Suppl. Fig.4


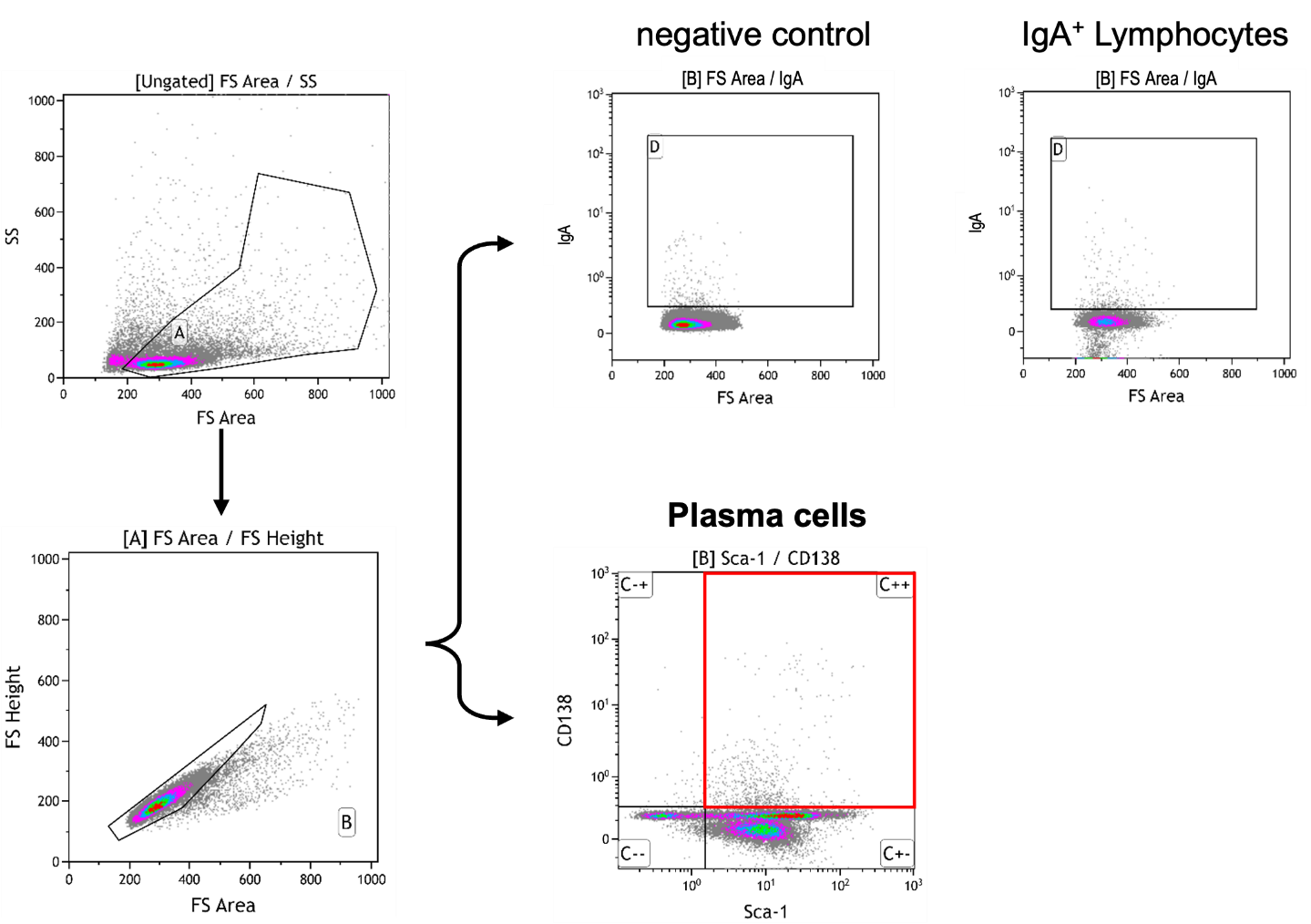


Suppl. Fig 4: Gating for IgA specific B cells and Plasma cells
